# Effect of a probiotic on blood pressure in grade 1 hypertension (HYPRO): protocol of a randomized controlled study

**DOI:** 10.1186/s13063-020-04973-0

**Published:** 2020-12-29

**Authors:** Anja Mähler, Nicola Wilck, Geraldine Rauch, Ralf Dechend, Dominik N. Müller

**Affiliations:** 1 Experimental and Clinical Research Center, a cooperation between Charité - Universitätsmedizin Berlin and Max Delbruck Center for Molecular Medicine, Berlin, Germany; 2 Charité - Universitätsmedizin Berlin, Freie Universität Berlin, Humboldt-Universität zu Berlin, and Berlin Institute of Health, Berlin, Germany; 3 Berlin Institute of Health, Berlin, Germany; 4 DZHK (German Centre for Cardiovascular Research) partner site Berlin, Berlin, Germany; 5 Max Delbrück Center for Molecular Medicine in the Helmholtz Association, Berlin, Germany; 6 Medizinische Klinik mit Schwerpunkt Nephrologie und Internistische Intensivmedizin Charité - Universitätsmedizin Berlin, Berlin, Germany; 7 Institute of Biometry and Clinical Epidemiology, Charité - Universitätsmedizin Berlin, Freie Universität Berlin, Humboldt-Universität zu Berlin, and Berlin Institute of Health, Berlin, Germany; 8 Department of Cardiology and Nephrology, HELIOS-Klinikum, Berlin, Germany

**Keywords:** Hypertension, Probiotic, Vivomixx®, Blood pressure, Microbiome, Metabolome, Immune cell phenotype, Glucose variability, Randomized controlled trial

## Abstract

**Background:**

Arterial hypertension is a major risk factor for cardiovascular disease and leads to target organ damage including stroke, heart failure, and kidney disease. About 1.5 billion people worldwide have hypertension, and it is estimated that it causes about 8 million deaths each year. Although there are several drugs available to lower blood pressure (BP), a great proportion of treated patients does not reach recommended treatment targets. Typical antihypertensive drugs target the vessels, the kidneys, and the heart. However, our gut microbiota also influences cardiovascular health, and gut dysbiosis is associated with hypertension. In this study protocol, we investigate the potential BP-lowering effect of a probiotic in patients with grade 1 hypertension.

**Methods:**

This study is an exploratory, randomized, double-blind, placebo-controlled, parallel-group study. One hundred ten patients with grade 1 hypertension (treated or untreated) will be randomized to either the probiotic Vivomixx® or placebo. The primary endpoint is the nocturnal systolic BP measured by ambulatory blood pressure monitoring after 8 weeks adjusted for the baseline value. The secondary endpoints are changes from baseline in nocturnal diastolic BP, antihypertensive medication, fecal microbiome composition, fecal and serum metabolome, immune cell phenotypes, glucose variability after three standardized breakfasts, and health-related quality of life (PROMIS-29). We also assess the safety profile of the intervention.

**Discussion:**

We postulate that various administrated bacteria (*Lactobacilli*, *Bifidobacteria*, and *Streptococcus thermophilus*) convert dietary components into active metabolites that positively affect immune cell function. A reduction of pro-inflammatory immune cell function could promote a BP-lowering effect.

**Trial registration:**

ClinicalTrials.gov NCT03906578. Registered on 08 April 2019

**Supplementary Information:**

The online version contains supplementary material available at 10.1186/s13063-020-04973-0.

## Introduction

Arterial hypertension is one of the major risk factors for cardiovascular disease, including ischemic heart disease, heart failure, kidney disease, and stroke. About 1.5 billion people worldwide develop arterial hypertension [1] to which about 8 million premature deaths per year are attributable [2]. The Global Burden of Disease Study 2013 revealed that—after dietary risks—high systolic blood pressure (BP) was the second most important risk factor for morbidity and mortality globally, exceeding the risks of tobacco smoke, high body mass index, high fasting plasma glucose, and high total cholesterol [3]. In 2017, 15% of women and 25% of men in Germany had elevated BP [4].

Commonly used antihypertensive drugs typically target different hormone systems (e.g., renin-angiotensin system) and the production of endogenous vasoactive substances, aiming to protect the blood vessels, heart, and kidney. However, despite the fact that there are several drugs available to lower BP, a large proportion of treated patients do not reach the BP targets recommended by current guidelines [5]. Non-pharmacological approaches to prevent and treat hypertension are lifestyle modifications, such as increased physical activity, reduced salt and alcohol intake, weight loss, and a healthy diet [6].

Another non-pharmacological approach to lower BP is the use of probiotics. Research efforts in this field first focused on milk fermented with *Lactobacillus helveticus*, which contains two tripeptides that inhibit the angiotensin-converting enzyme [7]. Since 1996, a number of clinical studies that investigated the BP-lowering effects of fermented dairy products in hypertensive subjects were published, and the majority of them showed BP-lowering effects. However, a Cochrane review in 2012 evaluated these studies and concluded that the effect on systolic BP was too modest to recommend a general use in the treatment of hypertension [8]. Two years later, a meta-analysis of 9 studies, which investigated the effect of various probiotics, found a modest but clinically meaningful BP reduction [9]. This was confirmed by a very recent meta-analysis of 14 studies published between 2002 and 2019 [10].

Here, we use a probiotic formulation with a high concentration of eight different live bacterial strains. Our proposed mode of action is not through angiotensin-converting enzyme inhibition but through the modulation of the microbiota-immune interaction. The gut microbiota is thought to be relevant for cardiovascular health [11], and microbial imbalance is linked to experimental and human hypertension [12, 13]. This is supported by recent epidemiological evidence showing that higher total yogurt intake lowers the risk of incident high BP in middle-aged and older adults [14]. Furthermore, hypertensive subjects with a high yogurt intake had a lower risk of developing cardiovascular disease [15].

Microbial metabolites are resorbed from the intestinal lumen and affect various functions of the host. Their effects are not restricted to the intestine but also affect the immune system, the vasculature, and the kidney. Hypertensive organ damage seems to be largely mediated by immune cells like effector memory T cells [16] and T cells producing interleukin-17 (Th17 cells) [17]. Recently, we demonstrated that a high-salt diet affected fecal *Lactobacillus* abundance in mice and humans alongside increased BP and Th17 abundance. A probiotic *Lactobacillus* treatment reduced the salt-induced increase in BP and Th17 [18]. In an exploratory study, we found that Vivomixx® prevented a high salt-induced increase in BP in healthy men (unpublished data, NCT02971787). In subjects eating a synbiotic yogurt for 90 days, we found that an increased capacity for intestinal short-chain fatty acid production was correlated with diastolic BP reduction [19].

Here, we present the protocol of a randomized, double-blind, placebo-controlled study investigating the effects of a probiotic in patients with grade 1 hypertension. We hypothesize that the probiotic is superior to placebo in lowering BP. Secondary endpoints are changes in antihypertensive medication, fecal microbiome composition, fecal and serum metabolome, immune cell phenotypes, glucose variability after three standardized breakfasts, and health-related quality of life.

## Methods

### Study design

This is an exploratory, randomized, double-blind, placebo-controlled, parallel-group study conducted at the Experimental and Clinical Research Center of Charité - Universitätsmedizin Berlin, Germany. Recruitment started in August 2019 (ClinicalTrials.gov identifier: NCT03906578). Patients are recruited from greater Berlin, Germany. Recruitment is performed via the hypertension outpatient clinic at Helios Klinikum Berlin-Buch, study flyers in medical practices, and advertisements in a broad range of media relevant to the study population. We intend to randomize 110 patients with grade 1 hypertension to either 8 weeks of Vivomixx® or placebo. The intervention is followed by a 4-week recovery period.

This study protocol was approved by the institutional review board of Charité - Universitätsmedizin Berlin, and we obtain written informed consent from all participants before study entry (Additional files 1 and 2). The study is conducted in accordance with the Declaration of Helsinki in its currently applicable version, the guidelines of the International Conference on Harmonization of Good Clinical Practice (ICH-GCP), and applicable German laws.

### Participants

Information and informed consent forms have been prepared in accordance with the guidelines of the institutional review board of Charité - Universitätsmedizin Berlin. Potential participants receive both forms at least 1 day before their screening visit. During this visit, a study physician explains all study procedures, and written informed consent is only given after participants had adequate time to ask questions. We include men and postmenopausal women with grade 1 hypertension in this study.

### Complete list of inclusion criteria

- Men and postmenopausal women (ratio 1:1)
- 50–75 years of age
- Grade 1 hypertension, defined as resting office blood pressure of 140–159 and/or 90–99 mmHg according to the European Societies of Cardiology and Hypertension (ESC/ESH) guidelines [20]
- Hypertension can be treated or untreated
- Body mass index between 18.5 and 34.9 kg/m^2^

### Complete list of exclusion criteria

- Secondary causes of hypertension
- Known target organ damage
- Pharmacological BP treatment which has been initiated recently (< 1 month) and is still in the optimization phase
- Ten-year cardiovascular risk score > 5% according to the 2017 ACC/AHA guideline
- Clinically relevant metabolic, autoimmune, chronic, progressive, or malignant diseases
- Postoperative phase
- Current or chronic infections
- Use of antibiotics within 3 months before the study
- Regular use of probiotics and dietary supplements
- Special diet for medical reasons
- Food intolerances and food allergies
- Vegan diet
- Weight loss diet or loss of more than 2 kg within 1 month before the study
- Inability to fully understand the significance, procedures, and scope of this study
- Known alcohol or drug abuse
- Participation in another interventional study

The discontinuation criteria are withdrawal of consent, subsequent occurrence of an exclusion criterion (e.g., change of BP medication), lack of compliance, and other medical reasons for stopping the intervention.

An advantage in terms of compliance is the fact that patients only have to take a single dose of the probiotic or placebo for a relatively short period. A potential disadvantage is the fact that they only suffer from a mild form of hypertension, which might decrease compliance. To improve and/or ensure compliance, we ask patients before inclusion if they intend to be compliant, establish a good relationship, give simple and precise verbal and written instructions, and directly question patients for regular probiotics/placebo intake and estimate compliance by counting unused sachets. These efforts also improve the retention of patients in the study. In addition, we will detect probiotic bacterial strains in stool samples. However, these bacteria are thought to transiently colonize the intestine. Thus, their detection in stool samples after 8 weeks of intervention does not necessarily confirm rigorous compliance.

### Randomization

Eligible patients are included after the successful screening. Randomization is done by the supplier (see below) based on the lists generated with an ad hoc SAS® program (sex stratification, randomly varying block sizes). Based on these lists, the supplier delivers sequentially numbered boxes containing sachets to the study center without any information on group allocation. Thus, strictly concealed allocation and complete blinding are ensured. A person of trust from the study center not involved in the study has access to sealed envelopes for each participant, thereby enabling disclosure of group allocation if necessary.

### Probiotic intervention

Vivomixx® is a microbiotic food supplement. It contains eight strains of live bacteria (*Lactobacilli paracasei*, *plantarum*, *acidophilus*, and *delbrueckii*; *Bifidobacteria longum*, *infantis*, and *breve*; *Streptococcus thermophilus*) in a concentration of 450 billion bacteria per sachet. Patients take the content of two sachets dissolved in water in the evening (approx. every 24 h, either 30 min before or 2 h after dinner). Two sachets yield a daily dose of 9 × 10^11^ colony-forming units (CFU). The placebo group takes sachets with an identically appearing powder without any bacteria. Both interventional products are provided by the supplier Mendes S. A, Lugano, Switzerland.

### Adverse events

Vivomixx® has been safely used in other studies and was well tolerated, even in patients with irritable bowel syndrome [21]. Minor adverse events of probiotics can be increased gas, bloating, and stool frequency. These side effects are mild and transient. Nevertheless, we question all patients at each visit for related or unrelated health issues and record all adverse events. Serious adverse events due to this probiotic are not to be expected.

### Outcome parameters

All outcomes are assessed at three time points—at baseline, after 8 weeks of intervention, and 4 weeks after discontinuation (recovery).

Our primary hypothesis is that a probiotic containing eight different strains of live bacteria reduces BP in grade 1 hypertension. Accordingly, our primary endpoint is the mean nocturnal systolic BP after 8 weeks of probiotic intake compared to placebo and adjusted for the baseline value. We already used this outcome to attain a maximally standardized way of BP measurement [18]. Other established outcomes in hypertension research like office BP and ambulatory 24-h BP will be included as important secondary outcomes.

Further secondary endpoints are change of antihypertensive medication, fecal microbiome composition, fecal and serum metabolome, immune cell phenotypes, glucose variability after three standardized breakfasts and health-related quality of life from baseline.

We perform a 4-week follow-up after the intervention, in which procedures will be repeated to investigate any prolonged treatment effects. We will also control for BP-reducing lifestyle factors such as weight reduction (anthropometry) and dietary habits including salt intake (food records) before and after the intervention.

Body composition is measured by air-displacement plethysmography, and the blood is sampled for routine and research measurements as well as peripheral blood mononuclear cell (PBMC) isolation. In addition, patients rate their health-related quality of life with the PROMIS-29 questionnaire.

Glucose variability is measured by continuous glucose monitoring (FreeStyle Libre Pro, Abbott). For this, a sensor is placed in the adipose tissue (triceps region) where it measures the interstitial glucose concentration every 15 min for 7 days. To study possible changes in postprandial glucose concentrations, patients are asked to eat three standardized breakfasts on three consecutive mornings while wearing the sensor. These breakfasts are prepared in the study center and handed out to patients to eat at home. Breakfasts are similar in calorie and carbohydrate content but differ in glycemic index (high, medium, low).

To evaluate and control for dietary intake, patients record food and beverage consumption in 4-day food records while wearing the sensor. Although food records over the whole 7 days might have been preferable, 4-day records are considered to yield sufficient results, are less demanding on patients and investigators, and reduce the time in which dietary habits might change due to recording, e.g., undereating. Records will be analyzed for macro- and micronutrient content using OptiDiet Plus (V6.0, GOE, Linden, Germany). This professional analysis software uses the nutrient content of 12,000 foods provided by the German Nutrient Database.

From stool samples, bacterial DNA will be sequenced, and fecal and serum metabolites will be measured by mass spectrometry. We will explore metabolites with –omics-based methods. We hypothesize that changes in the gut microbiota affect the ratio of certain tryptophan metabolites and levels of short-chain fatty acids, which in turn affect immune responses and blood pressure. However, we will also design more experiments that address the biological question of this study. The obtained results will enable the construction of rigorous scientific hypotheses which can be tested later using -omic or non-omic methods.

Table 1 provides a detailed overview of assessments and endpoints.

**Table 1:** Schedule of HYPRO study visits

Study visit	V0	V1	V2	V3
Time point	Screening	Baseline	Post-intervention	Recovery
Week	− 4 to − 1	0	8	12
Informed consent	x			
Inclusion/exclusion criteria	x			
Demographics	x			
Case history	x			
Medication	x	x	x	x
Physical exam	x			
Smoking status	x			
Height, weight, waist-to-hip ratio	x			
Blood pressure, heart rate, ECG	x			
Blood sample	x	x	x	x
Urine sample	x	x	x	x
AE/SAE query		x	x	x
Bodyweight and composition (ADP)		x	x	x
Office BP		x	x	x
24-h ABPM		x	x	x
PBMC isolation		x	x	x
Glucose sensor (7 days)		x	x	x
Food record (4 days)		x	x	x
Standardized breakfasts (3 days)		x	x	x
Stool sample		x	x	x
Health-related quality of life (PROMIS-29)		x	x	x

*ABPM* ambulatory blood pressure monitoring, *ADP* air-displacement plethysmography, *AE* adverse event, *BP* blood pressure, *ECG* electrocardiogram, *PBMC* peripheral blood mononuclear cells, *PROMIS* Patient-Reported Outcomes Measurement Information System, *SAE* serious adverse event

### Power calculation

Based on the above mentioned meta-analysis [9] and on our own unpublished data (NCT02509962, NCT02971787), we estimate a mean decrease in systolic BP of 3.8 mmHg with a standard deviation of 5.0 mmHg in the probiotic group. In addition, we assume that the placebo group will have the same systolic BP baseline value and a small decrease in the mean systolic BP of 0.2 mmHg with the same standard deviation. That leads to a standardized effect size between groups of (3.8 − 0.2)/(√2 × 5.0) ≈ 0.51. The required sample size to find a significant effect with a power of 0.8 at a two-sided significance level of 0.1 is given by 98 patients (49 per treatment arm, calculated with ADDPLAN, version 6.1). To account for approximately 10% dropout, the total number of patients to be recruited is 110 (98/110 ≈ 0.9).

### Data management

To protect participant’s confidentiality, only pseudonyms are used for all data documentation, sample markings, and communication with third parties (e.g., laboratories, collaboration partners). Clinical data are collected in source documents and electronic case report forms (Research Electronic Data Capture database). Database entries are automatically checked for data format and appropriate range. We do not rely on an additional independent monitoring for this is a single-center, exploratory study. Due to the minimal risks of the probiotic intervention, a data monitoring committee is deemed unnecessary. We will submit modifications to this protocol to the institutional review board, participants, and investigators. After completion of the study, data will be stored for 10 years and then be deleted.

We do not ask participants for broad consent. Thus, we will only use data and samples for the hypotheses stated in this protocol and will not provide them for ancillary studies.

After completion of this study, the results will be expounded to participants, presented on scientific conferences, published in peer-reviewed journals, and circulated to physicians and the medical laity. We will comply with the official eligibility guidelines for authorship for all publications and do not intend to use professional writers.

### Data analysis

A confirmatory analysis will be conducted based on the intention-to-treat (ITT) population, defined by the ITT principle. We want to show that the intervention group is superior to the control, i.e., the mean nocturnal systolic BP at 8 weeks after the start of treatment adjusted for the baseline value is lower in the intervention group than in the control group. An analysis of covariance (ANCOVA) adjusted for baseline systolic BP values using group allocation as a factor will be applied. The global two-sided significance level is 0.1. Missing values will be replaced using multiple imputation [22].

A sensitivity analysis to the primary efficacy ANCOVA model will be applied with additional covariates/factors given by age and sex. Subgroup analysis is planned for sex. Descriptive methods will be used for the analysis of all clinical, demographic, and safety parameters, including the calculation of appropriate summary measures of the empirical distribution as well as 95% confidence intervals and calculation of descriptive two-sided *p* values. A safety analysis of frequencies and rates of adverse events will be performed. Additionally, sensitivity analyses will be conducted for different populations (per-protocol population, patients with complete cases). Interim analyses are not included in our analysis plan. All analyses will be done using validated statistical software.

## Discussion

With this study, we want to close the gap between the existing epidemiological, experimental, and our own clinical data in healthy volunteers. We will do this by thoroughly investigating the effects of a probiotic that has not been used in hypertensive patients before. However, we do not only aim at alleviating hypertension, we will also use state-of-the-art omics systems approaches to understand the impact of probiotics on the gut microbiome, immunome, metabolome, and vascular phenotypes in these patients. To the best of our knowledge, this is the first study that comprehensively investigates all of these levels in human hypertension.

We hypothesize that targeting the intestinal microbial community with live bacteria will improve hypertension along with an improved immune cell homeostasis. This mode of action could also be effective in treated hypertensive patients who do not reach the desired treatment targets. An improved immune homeostasis could not only lead to lower BP but also to better target organ protection, since immune cells are known to migrate into target organs thereby promoting organ dysfunction. Further, probiotic strains might stably colonize the gut and might evoke—in contrast to classical pharmacological therapy—a long-lasting antihypertensive benefit after discontinuation of treatment. This will be tested in our follow-up phase. Moreover, probiotics are generally well tolerated and have little side effects. Thus, compliance in hypertensive patients, who are skeptical of classical pharmacotherapy, might be improved.

We include either untreated grade 1 hypertensive patients in whom pharmacological treatment can be postponed for a limited time in favor of lifestyle modifications or treated patients who are not on goal despite adequate pharmacotherapy. Included patients have no clinical signs of target organ damage, serious concomitant diseases, or secondary causes of hypertension. From our point of view, these mildly hypertensive patients would benefit most from a probiotic intervention. Furthermore, according to applicable guidelines, it is possible to delay the start or change of pharmacotherapy for the 12-week study duration at this stage of the disease. This choice will limit the extrapolation of our results to other hypertension grades, such as patients with resistant hypertension. However, these patients have a higher risk for cardiovascular events, and necessary therapeutic changes cannot be postponed.

Interventional considerations were based on a meta-analysis that suggested an intervention period of ≥ 8 weeks with ≥ 10^11^ CFU/day of a multi-strain probiotic to effectively lower BP [9].

We chose nocturnal BP instead of office BP or 24-h ambulatory BP measurement to achieve a highly standardized measurement in the patient’s natural environment. This standardized measurement, which excludes variations due to physical activity during the day, allows to conduct this study with a medium sample size.

Although there is a high level of interest among eligible patients, recruitment turned out to be rather challenging. This is mainly due to the fact that BP is highly variable within subjects. About 8 of 10 candidates fail our pre-screening, because they cannot be classified as grade 1 hypertensive. Recruitment is additionally impeded by common comorbidities, such as insulin-dependent type 2 diabetes or severe obesity. Nevertheless, due to our medium sample size, it is important that demographic, cardiovascular, and metabolic characteristics are well-defined and as narrow as possible.

All biomaterials, in particular, stool samples, are collected with the highest scientific standards to allow for high-quality microbiome analyses [23].

In conclusion, probiotic treatment of patients with grade 1 hypertension has the potential to lower BP. Due to its innovative mode of action, probiotic treatments could be considered for prevention, might delay pharmacotherapy, and enable dose reduction of antihypertensive drugs, and thereby contribute to target organ protection and the reduction of cardiovascular events.

### Trial status

This article is based on the study protocol version 1.4 of 2 July 2019. Recruitment started on 28 August 2019 and will probably continue until March 2022.

## Supplementary Information

**Additional file 1.** : Approval of the institutional review board of Charité - Universitätsmedizin Berlin.

**Additional file 2.** : Written informed consent form.

## Data Availability

Not applicable.

## Abbreviations

ABPM: Ambulatory blood pressure monitoring
ADP: Air-displacement plethysmography
AE: Adverse event
BP: Blood pressure
CFU: Colony-forming units
ECG: Electrocardiogram
ESC: European Society of Cardiology
ESH: European Society of Hypertension
PBMC: Peripheral blood mononuclear cells
PROMIS: Patient-Reported Outcomes Measurement Information System
SAE: Serious adverse event
