# Supplementary material for: Effect of a probiotic on blood pressure in grade 1 hypertension (HYPRO): protocol of a randomized controlled study
Source: Trials. 2020 Dec 29;21:1032. doi: 10.1186/s13063-020-04973-0 (PMC7771080; doi:10.1186/s13063-020-04973-0)
Supplement: Supplementary file 2 — Additional file 2. : Written informed consent form. [file 13063_2020_4973_MOESM2_ESM.pdf]

Charité | Campus Buch | Lindenberger Weg 80 | 13125 Berlin

Dr. med. Michael Boschmann
Franz-Volhard-Centrum für Klinische Forschung am Experimental & Clinical Research Center - ECRC - eine Kooperation mit dem MDC
Direktor: Univ.-Prof. Dr. Friedemann Paul
Lindenberger Weg 80
13125 BERLIN

Tel. +49(0)30 450 540 241
Fax +49(0)30 450 540 920
michael.boschmann@charite.de
www.mdc-berlin.de/probanden

Berlin, 28.06.2019

Einwilligungserklärung

Einfluss eines Probiotikums auf den Blutdruck von Patienten mit Hypertonie Grad 1 - eine randomisierte klinische Studie (HYPRO-Studie)

Hiermit erkläre ich,

Vorname: ______________________________ Nachname: ____________________________

Adresse: ____ Geburtsdatum: _______________

Telefonnummer: ____

E-Mailadresse: ____

dass ich durch Herrn/Frau ....

(Name des Studienarztes / der Studienärztin)

mündlich und schriftlich über das Wesen, die Bedeutung und die Risiken der wissenschaftlichen Untersuchungen im Rahmen der o.g. Studie informiert wurde und ausreichend Gelegenheit hatte, meine Fragen mit dem Studienarzt / der Studienärztin zu klären.

Mir ist bekannt, dass ich das Recht habe, meine Einwilligung jederzeit ohne Angabe von Gründen und ohne nachteilige Folgen für mich zurückzuziehen und einer Weiterverarbeitung meiner Daten und Proben widersprechen und ihre Vernichtung verlangen kann.

Ich habe eine Kopie der schriftlichen Studieninformation und der Einwilligungserklärung mit Versions-Datum ____________________ erhalten.

Ich erkläre, dass ich freiwillig bereit bin, an der wissenschaftlichen Studie teilzunehmen.

Ich erkläre mich damit einverstanden,

1. dass meine für den Zweck der o.g. Studie nötigen personenbezogenen Daten wie in der Studieninformation vom ______________________ beschrieben durch den Studienarzt erhoben und pseudonymisiert aufgezeichnet und verarbeitet werden, auch auf elektronischen Datenträgern;

2. dass die Studienergebnisse in anonymer Form, die keinen Rückschluss auf meine Person zulässt, veröffentlicht werden;

3. dass die für den Zweck der o.g. Studie entnommenen Blutproben, pseudonymisiert durch den Studienarzt, im Labor *Labor Berlin – Charité Vivantes GmbH, Sylter Str. 2, 13353 Berlin* **untersucht und 10 Jahre gelagert werden können;**

4. dass für den Zweck o.g. Studie meine pseudonymisierten Studiendaten übermittelt werden an *Prof. G. Rauch, Institut für Biometrie und Klinische Epidemiologie, Charité.*

Berlin, den _________________ Unterschrift des Teilnehmers ___________________________

Hiermit erkläre ich, den/die Teilnehmer/in am ……………….... über Wesen, Bedeutung und Risiken der o.g. Studie mündlich und schriftlich aufgeklärt, alle Fragen beantwortet und ihm/ihr eine Kopie der Studieninformation und der Einwilligungserklärung übergeben habe.

Berlin, den _________________ Unterschrift des aufklärenden Studienarztes _______________
