# Supplementary material for: Effect of a probiotic on blood pressure in grade 1 hypertension (HYPRO): protocol of a randomized controlled study
Source: Trials. 2020 Dec 29;21:1032. doi: 10.1186/s13063-020-04973-0 (PMC7771080; doi:10.1186/s13063-020-04973-0)
Supplement: Supplementary file 1 — Additional file 1. : Approval of the institutional review board of Charité - Universitätsmedizin Berlin. [file 13063_2020_4973_MOESM1_ESM.pdf]

Charité | 10117 Berlin

Ethics Committee
Ethics Committee at the Charité Mitte Campus

Chair: Prof. Dr. R. Morgenstern

Managing directors: Dr. Katja Orzechowski
ethikkommission@charite.de

Correspondence address: Chariteplatz 1, 10117 Berlin, Germany
Tel.: +49 (0)30 450 517 222
Fax: +49 (0)30 450 517 952

http://ethikkommission.charite.de

To
Dr. Michael Boschmann
Charité – University Medical Center Berlin
Franz Volhard Clinical Research Center at the
Experimental & Clinical Research Centre
Lindenberger Weg 80
13125 Berlin, Germany

Cc: anja.maehler@charite.de

Date: 07/02/2019

Effect of a Probiotic on Blood Pressure in Low-to-moderate Risk Grade 1 Hypertension – a Randomized Study (HYPRO)
Proposal number: EA1/237/18
Processed on 06/28/2019, received on 07/02/2019, via e-mail on 06/28/2019

Dear Dr. Boschmann,

We hereby confirm receipt of the letter dated 06/28/2019 with the following attachments:

- Application for ethical approval, version 4 of 06/28/2019
- Patient information, version 4 of 06/28/2019
- Declaration of informed consent, version 4 of 06/28/2019

Thank you for notifying us. The ethics committee raises no objections to the amendments.

Sincerely,

[signature]

Prof. Dr. R. Morgenstern
Chair
